# Sex- and subregion-specific vulnerability of the hippocampus and amygdala to intermittent hypoxia in relation to learning/memory function and anxiety tendencies of infant rats

**DOI:** 10.1007/s11325-025-03382-4

**Published:** 2025-06-09

**Authors:** Yu Takenouchi, Jun Hosomichi, Kitanon Angkanawaraphan, Hideyuki Maeda, Haixin Hong, Chidsanu Changsiripun, Takashi Ono

**Affiliations:** 1 Department of Orthodontic Science, Graduate School of Medical and Dental Sciences, Institute of Science Tokyo, Tokyo, Japan; 2 Department of Forensic Medicine, Graduate School of Medicine, Tokyo Medical University, Tokyo, Japan; 3 Department of Forensic Medicine, Graduate School of Medicine, Osaka University, Osaka, Japan; 4 Shenzhen People’s Hospital, Shenzhen, China; 5 Department of Orthodontics, Faculty of Dentistry, Chulalongkorn University, Bangkok, Thailand

**Keywords:** Intermittent hypoxia, Cognitive function, Anxiety tendency, Rats, Hippocampus, Amygdala

## Abstract

**Purpose:**

Intermittent hypoxia (IH) is associated with learning/memory dysfunction during the early growth period. However, the sex- and subregion-specific brain vulnerability to IH and its effects on learning, memory, and emotional stability in infants remain unclear. This study investigated sex- and subregion-specific vulnerability to IH in the hippocampus, relating to memory and learning, and in the amygdala, relating to early emotional development in infant rats.

**Methods:**

Thirty-six 1-week-old Sprague-Dawley rats were exposed to IH (IH group) or normoxic air (N group). Learning/memory functions, emotional behavior, and locomotor activity were examined using the Y-maze apparatus, passive avoidance, and open field tests. The hippocampal cornu ammonis (CA) 1 and CA3 regions, dentate gyrus (DG), and amygdala were examined to measure *Ntrk2, Hif1a,* and *Epas1* expressions. A two-way analysis of variance followed by Tukey–Kramer’s honestly significant difference post-hoc analysis, or non-parametric equivalents and independent t-test were used to assess the data.

**Results:**

IH exposure negatively regulated long-term spatial memory and anxiety in male and female rats and short-term spatial memory in male rats. IH effects on brain development were validated by the increased expression of *Ntrk2* and *Epas1* mRNA in the DG, *Ntrk2* and *Hif1a* mRNA in the amygdala, and an increase in the immunohistochemically stained areas in the DG and amygdala of only male rats.

**Conclusion:**

These findings provide *in vivo* evidence for sex- and subregion-specific functional linkages between cognitive function and IH, and between anxiety tendency and IH during the early growth period.

## Introduction

Pediatric obstructive sleep apnea (POSA) primarily leads to neurocognitive underdevelopment and emotional immaturity [1]. Intermittent hypoxia (IH), a hallmark of POSA, involves repetitive deoxygenation-reoxygenation cycles, causing low blood oxygen saturation during sleep [2]. Research has shown a positive link between the apnea-hypopnea index and learning/memory deficits [3]. Rodent POSA models demonstrate that IH triggers neuronal apoptosis in developing brains [4, 5], indicating the hippocampus's heightened vulnerability to IH in early life [5].

The hippocampus, crucial for memory consolidation and learning, consists of distinct subregions with diverse neuronal cell types, including granule cells in the dentate gyrus (DG) and pyramidal cells in the cornu ammonis (CA), which vary in morphology and vulnerability to neuropathological conditions [6]. IH elevates apoptotic cells in a young rat’s hippocampus, leading to memory and learning deficits [4]. Consequently, the hippocampal susceptibility to neuronal injury under IH is area-dependent [7], affecting cognitive function during development based on specific hippocampal subregions.

Proposed mechanisms for IH-induced neuronal apoptosis and neurocognitive impairment include oxidative stress and dysregulation of the brain-derived neurotrophic factor (BDNF)/tyrosine kinase receptor B (TrkB) signaling pathway [8]. The BDNF/TrkB pathway is crucial for neuronal development in the immature central nervous system, including the hippocampus [9]. In rodents, dysregulated BDNF/TrkB signaling and the mitogen-activated protein kinase pathways impair memory/learning and reduce neuronal cells in the CA1 and CA3 hippocampal regions [10, 11]. Xie et al. [12] found that IH exposure in mice decreased hippocampal neuronal excitability and mature BDNF levels, while BDNF microinjection into the brains of hypoxic mice prevented neuronal excitability impairment [12]. However, few studies have linked BDNF expression to memory/learning function via behavioral tests.

Neonatal hypoxemia results in long-term grey matter reduction in the amygdala of children, which correlates with the onset of bipolar disorder [13]. This indicates that IH negatively affects emotional processing through amygdala disturbances. Functional magnetic resonance imaging studies show that altered amygdala function impairs emotional processing in bipolar disorder patients [14]. Animal studies reveal significant long-term loss of adrenocorticotropin-releasing factor-positive neurons, reduced cell body size, and axonal degeneration in the amygdala following prenatal and perinatal hypoxia [15]. These changes are linked to behavioral abnormalities, such as increased spontaneous locomotion and exploratory behavior [15]. It is suggested that hypoxia and ischemia-associated alterations may lead to long-term structural changes in the amygdala and impaired emotional development [13].

Despite structural changes in the hippocampus of growing IH rodents, the underlying mechanisms remain unclear. Neuroplasticity in response to IH shows sex differences in rodent models [16], with increased Fosb/ΔFosb-positive cells and behavioral impacts only in male rats. For instance, in response to IH, ovariectomized mice show impaired spatial learning and memory compared to other female groups, while intact male mice subjected to IH also exhibit impaired learning and memory compared to intact or castrated males exposed to room air [17].

Gestational hypoxia exposure induces anxiety-like behaviors only in male rat pups, suggesting greater male amygdala vulnerability to IH [18]. We hypothesized that sex- and subregion-specific brain vulnerability to IH impairs learning, memory, and emotional stability in infant rats. However, sex differences in the amygdala’s response to IH and sex-specific amygdala vulnerability remain unexplored. In this study, we aimed to investigate sex- and subregion-specific hippocampal vulnerability to IH in memory/learning, and amygdala vulnerability to IH during early emotional development in infant rats.

## Materials and methods

### Animal model

Thirty-six 1-week-old Sprague-Dawley rats (18 males and 18 females) were utilized. The pups and their mothers were randomly divided into two groups: the experimental group (IH group), which experienced IH for 8 hours during the 12-hour"lights-on"period at a rate of 20 cycles per hour (oxygen levels fluctuating from 4–21% with 0% carbon dioxide) from postnatal days 8 to 21, and the control group (N group), which was exposed to normoxic air. Both groups were housed in identical plastic cages positioned adjacent to each other to ensure consistent noise levels and minimize noise-related stress [19]. Bromodeoxyuridine (BrdU) (100 mg/g of body weight) was injected intraperitoneally into all pups once a day for 3 days (postnatal days 18–20) to measure cell proliferation. Ethical and animal protocol approvals were obtained from the Institutional Animal Care and Use Committee of Tokyo Medical University (approval numbers: R1-0125, September 20, 2019; R2-0033, March 2, 2020).

### Behavioral tests

The Y-maze apparatus (Muromachi Kikai, Tokyo, Japan) comprises three equally angled arms (300 x 60 x 150 mm), designated A, B, and C. Rats (postnatal day 20) explored all arms for 8 minutes. Arm entries and triads were recorded to calculate alternation percentage [10, 11] using the formula:

$$\textrm{Percent}\ \textrm{alternation}\ \left(\%\right)=\left(\left[\textrm{number}\ \textrm{of}\ \textrm{alternation}\textrm{s}\right]/\left[\textrm{total}\ \textrm{arm}\ \textrm{entries}-2\right]\right)\times 100$$

An arm entry was defined as the placement of all four limbs within the arm.

The passive avoidance test apparatus, consisting of an illuminated and a dark compartment separated by a guillotine door, was used to assess memory. Rats were habituated to both compartments on postnatal day 19. During training on postnatal day 20, the time taken to enter the dark compartment, where they received an electrical stimulation, was recorded. After 24 hours, the time to re-enter the dark compartment was measured and compared to the initial time [10, 11].

An open field test recorded the spontaneous activity of the rats for 30 min in a square box (29.7 x 42.0 x 6.5 cm) using a digital camera [20]. Digital images were analyzed with EthoVision 3.0 (Noldus, Wageningen, Netherlands) as per the manufacturer's instructions. According to a previous study, rats were acclimated to the open field box for 10 min before monitoring [21]. The mean total distance travelled and the average time spent in the center zone between the 10 th and 20 th minutes of the session were used for group comparison.

### Preparation of histological sections

Regarding post-behavioral testing on postnatal day 21, rats were euthanized under isoflurane anesthesia. Brains were extracted, hemisected, and perfused with 4% paraformaldehyde. Tissues were then cryoprotected in 30% sucrose for 3 days, embedded in 5% super cryoembedding medium gel, and frozen in cold hexane. The frozen brain blocks were sectioned into 20-μm slices using a cryostat at −20 °C and mounted on RNase-free PEN-membrane glass slides.

### Immunohistochemical staining

Frozen sections were incubated overnight at 4 °C with primary anti-BrdU (1∶100, NB 500-235, Novus Biologicals, Centennial, CO, USA) and anti-BDNF antibodies (1∶100, bs-4989R; Bioss, Woburn, MA, USA). They were then treated with pre-diluted biotinylated secondary antibodies for 30 min, followed by VECTASTAIN Elite ABC Reagent (Vector Laboratories, Newark, CA, USA) for 30 min, and a peroxidase substrate solution for 1 min. Hematoxylin staining was performed afterward. Phosphate-buffered saline containing 1% bovine serum albumin was used as an isotype control. Image J software was used to measure the number of immunopositive cells and total stained area (μm^2^) within a 100 x 100 μm region of interest to evaluate protein expression in CA1, CA3, DG, and amygdala areas.

### Laser microdissection and quantitative polymerase chain reaction analysis

Tissue samples from the CA1, CA3, DG, and amygdala regions were isolated using LMD7000 (Leica Microsystems, Nussloch, Germany) and collected into 0.5-ml microtube caps, with three slides per site for quantitative polymerase chain reaction (qPCR) analysis. Total RNA was extracted using the PureLink FFPE RNA Isolation Kit (Thermo Fisher Scientific, Waltham, MA, USA) and converted to cDNA. mRNA levels of *Ntrk2, Hif1a*, and *Epas1* were quantified by real-time PCR (Applied Biosystems 7500, Thermo Fisher Scientific) using Probe qPCR Mix and TaqMan probes from Takara Bio (Otsu, Shiga, Japan) (Table 1), with *Actb* as the internal control. The comparative Ct method was used to compare mRNA expression levels in the IH group to the N group by sex.

**Table 1:** List of target genes quantified using real-time polymerase chain reaction

Target gene	Forward primer	Reverse primer
*Ntrk2*	5'-GAACCTCACTGTGCATTTTGCA-3'	5'-GCTCCGTTGTAGAACCACTGAA-3'
*Hif1a*	5'-CACGATCATATCACTGGACTTCG-3'	5'-CAGAGGCAGGTAATGGAGACA-3'
*Epas1*	5'-CTGTCAGAAAACATCAGCAAGTTC-3'	5'-TCTCGGATCTCCTCGTGGTC-3'
*Actb*	5'-GCTATGAGCTGCCTGACGGT-3'	5'-ATGCCACAGGATTCCATACCC-3'

### Statistical analysis

All statistical analyses were performed using JMP 10.0 (SAS Institute, Cary, NC, USA). Data were expressed as mean ± standard deviation (SD). Body weight, open field, and Y-maze tests were assessed using the two-way analysis of variance (ANOVA) with Tukey–Kramer’s honestly significant difference (HSD) post-hoc analysis or their non-parametric counterparts. An independent t-test was used to compare passive avoidance test results, relative gene expressions, and relative protein expressions between same-sex groups. Statistical significance was set at *p* < 0.05 for the t-test and *p* < 0.01 for ANOVA with Tukey’s HSD test.

## Results

### Body weight changes

On postnatal day 14, female IH rats showed a greater decrease in body weight compared to female N rats (Table 2). By postnatal day 21, significant weight differences were noted between N and IH groups in both sexes.

**Table 2:** Body weight changes in normoxic and intermittent hypoxia rats

Age	N male (*n* = 9)	N female (*n* = 9)	IH male (*n* = 9)	IH female (*n* = 9)
7-day-old	13.39 ± 1.19	14.39 ± 1.56	13.61 ± 1.41	14.00 ± 1.75
14-day-old	22.5 ± 3.82	22.61 ± 3.31	21.00 ± 0.97	19.50 ± 1.09 #
21-day-old	37.56 ± 1.24	35.67 ± 2.33	29.83 ± 3.48 **	27.61 ± 3.70 ##

Values are presented as mean ± standard deviation (n, numbers of rats)

*: *p* < 0.05 vs. N-male

**: *p* < 0.01 vs N male

^#^: *p* < 0.05 vs. N female

^##^: *p* < 0.01 vs. N-female

*IH* intermittent hypoxia, *N* normoxia

### Effect of IH on memory/learning function in infant rats

**Fig. 1:**
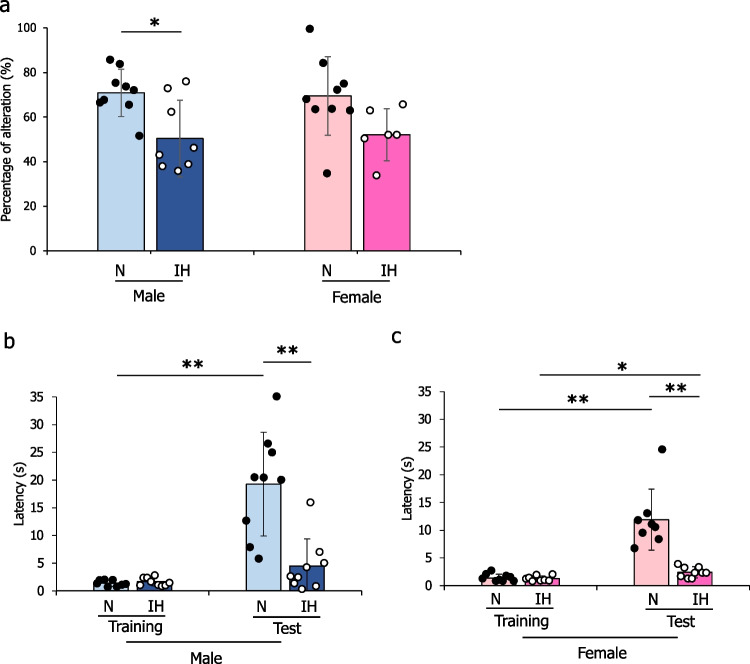
Mean percentage of alternation in the Y-maze test of male and female rats (**a**). Latency in the passive avoidance test of the normoxic and IH groups in (**b**) males (*n* = 9 each) and (**c**) females (*n* = 9 each). The data are presented as the mean ± standard deviation for each group. *: *p* < 0.05; **: *p* < 0.01. Abbreviations: IH, intermittent hypoxia; N, normoxia

A Y-maze test revealed a significant difference (*p* = 0.010) in alternation percentage between N (70.87±10.66%) and IH (50.32±17.59%) male rats, but no significant difference (*p* = 0.051) between N (69.46±17.22%) and IH (52.08±11.71%) female rats (Fig. 1a). In the passive avoidance test, there was a significantly increased latency from training in both the N male and female rats (N males, 1.38±0.59 s; N females, 1.38±0.68 s) to 24 hours after the training test (N males, 19.27±9.36 s; N females, 11.91±5.50 s) (Figs. 1b–c). However, IH males showed no significant change in latency between training (1.67±0.76 s) and testing (4.52±4.86 s) (Fig. 1b), while IH females exhibited a significant difference between training (1.31±0.46 s) and 24-hour testing (2.40±1.01 s) (Fig. 1c).

### Effect of IH on emotional development in infant rats

**Fig. 2:**
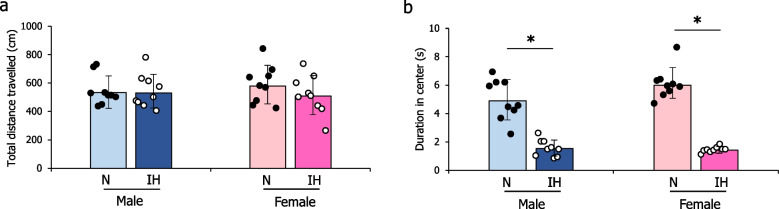
Total distance traveled (**a**) and amount of time spent in the center zone in the open-field box (**b**) between the 10 th and 20 th minutes of the monitoring session. The data are presented as the mean ± standard deviation for each group. *: *p* < 0.05; **: *p* < 0.01. Abbreviations: IH, intermittent hypoxia; N, normoxia

In the open-field test, there was no significant main effect of treatment or sex on total distance travelled, and the treatment-by-sex interaction was not significant (F1,32 = 0.69, *p* = 0.041) (Fig. 2a). However, a significant treatment effect on center zone time was found (F1,32 = 159.00, *p* < 0.001), with IH pups spending significantly less time in the center than N group pups (*p* < 0.001 for both sexes) (Fig. 2b). Sex effects and treatment-by-sex interactions for center zone time were not significant (F1,32 = 2.49, *p* = 0.125; F1,32 = 3.70, *p* = 0.06) (Fig. 2b).

### Histological analysis

**Fig. 3:**
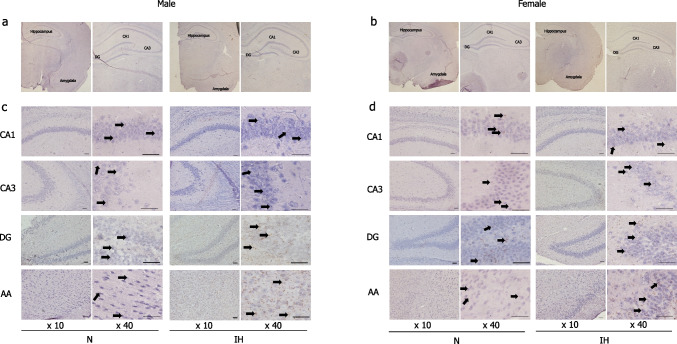
Expression pattern of BDNF in the hippocampus and amygdala of the infant rats after 7 days of IH exposure. Immunohistochemical staining images of the CA1, CA3, and DG of the hippocampus, as well as the amygdala. The hippocampus and amygdala regions of males (**a**). The hippocampus and amygdala regions of females (**b**). Magnified images of the hippocampal CA1, CA3, and DG regions and the amygdala region of males (**c**). Magnified images of the hippocampal CA1, CA3, and DG regions and the amygdala region of females (**d**). The arrows indicate positive areas for DAB staining. Scale bar represents 100 μm in the images. Abbreviations: BDNF, brain-derived neurotrophic factor; CA, cornu ammonis; DG, dentate gyrus; IH, intermittent hypoxia; N, normoxia; DAB, 3,3′-Diaminobenzidine

**Fig. 4:**
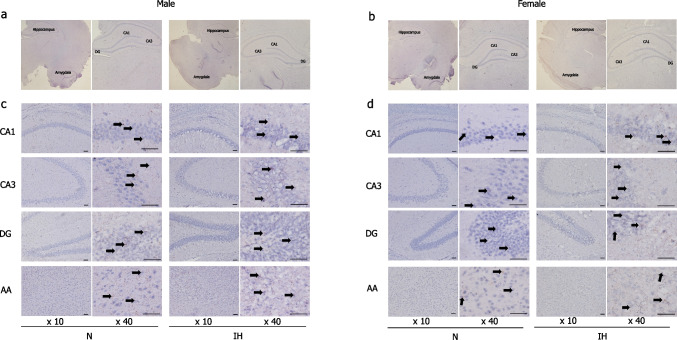
Expression pattern of BrdU in the hippocampus and amygdala of the infant rats after 7 days of IH exposure. Immunohistochemical staining images of the CA1, CA3, and DG of the hippocampus, as well as the amygdala. The hippocampus and amygdala regions of males (**a**). The hippocampus and amygdala regions of females (**b**). Magnified images of the hippocampal CA1, CA3, and DG regions and the amygdala region of males (**c**). Magnified images of the hippocampal CA1, CA3, and DG regions and the amygdala region of females (**d**). The arrows indicate positive areas for DAB staining. Scale bar represents 100 μm in the images. Abbreviations: BrdU, Bromodeoxyuridine; CA, cornu ammonis; DG, dentate gyrus; IH, intermittent hypoxia; N, normoxia; DAB, 3,3'-Diaminobenzidine

**Fig. 5:**
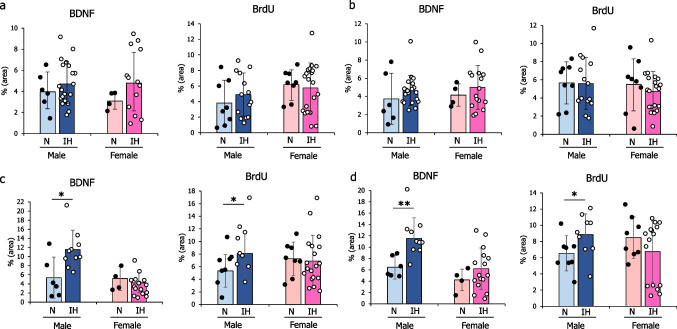
Quantification of immunopositive cell area in the hippocampus and amygdala of male and female rats. Ratio of immuno-positive cell area for BDNF and BrdU in (**a**) the CA1, (**b**) CA3, (**c**) dentate gyrus of the hippocampus, and (**d**) amygdala. The data are presented as the mean ± standard deviation for each group. *: *p *< 0.05; **: *p *< 0.01. Abbreviations: BDNF, brain-derived neurotrophic factor; BrdU, Bromodeoxyuridine; CA, cornu ammonis; IH, intermittent hypoxia; N, normoxia

We examined BDNF- and BrdU-immunopositive cells in the hippocampus and amygdala, key regions for memory and emotion regulation, by using sections that had been properly stained and encased in young rats exposed to IH. No significant morphological changes, such as atrophy, were observed in either region between the N and IH groups for both sexes (Figs. 3a–b and 4a–b). At high magnification, neuron size and cellular arrangement remained unchanged in both males and females (Figs. 3c–d and 4c–d). BDNF and BrdU expression levels were similar between IH and N groups in the CA1 (Fig. 5a) and CA3 (Fig. 5b) regions of both male and female rats. However, in male rats, IH significantly increased BDNF and BrdU expressions in the DG (2.15-fold, *p* = 0.01 in BDNF, and 1.52-fold, *p* = 0.04 in BrdU) (Fig. 5c) and the amygdala (1.78-fold, *p* = 0.002 in BDNF, and 1.35-fold, *p* = 0.03 in BrdU) (Fig. 5d). In contrast, in female rats, BDNF and BrdU levels remained comparable between the IH and N groups in the CA1 (Fig. 5a), CA3 (Fig. 5b), DG (Fig. 5c) of the hippocampus, and amygdala (Fig. 5d).

### Sex- and subregion-specific vulnerability of gene expressions to IH

**Fig. 6:**
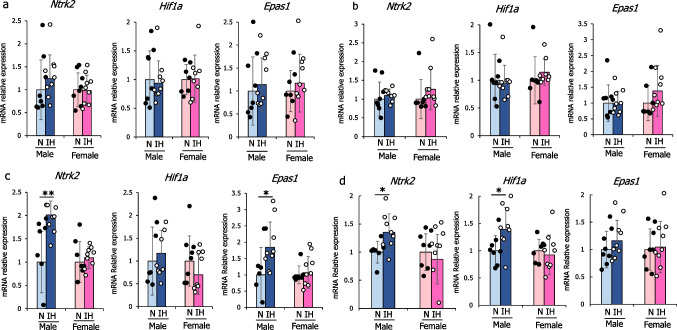
Relative mRNA expression levels of *Ntrk2*, *Hif1a*, and *Epas1* mRNAs in (**a**) the CA1, (**b**) CA3, (**c**) dentate gyrus of the hippocampus, and (**d**) amygdala of the male and female rats. The mRNA-expression levels measured in the normoxic rats were set to a value of 1. The data are presented as the mean ± standard deviation for each group. *: *p* < 0.05; **: *p* < 0.01. Abbreviations: CA, cornu ammonis; IH, intermittent hypoxia; N, normoxia

*Ntrk2*, *Hif1a*, and *Epas1* gene expressions were comparable in the CA1 and CA3 areas of infant male rats between the IH and N groups (Figs. 6a–b). Nevertheless, *Ntrk2* significantly rose in the DG and amygdala of IH male rats, with higher *Hif1a* mRNA in the amygdala and *Epas1* mRNA in the DG than in N male rats (Figs. 6c–d). In female rats, no notable differences in these gene expressions were detected across the CA1, CA3, DG, or amygdala between the IH and N groups.

## Discussion

Immunohistochemical and qPCR data suggest that the rat hippocampal DG region is particularly susceptible to IH in male infants. The DG region, which supplies neuronal fibers to the CA1 and CA3 regions [22], is positively linked with memory function and neurogenesis [23]. The BrdU-stained area increased only in the DG region of male rats, despite the usual correlation of CA1 and CA3 regions with neural cell proliferation. These regional differences may be due to temporal and spatial variations. The effect of IH on nerve fiber numbers in the adult hippocampus remains controversial. No significant gene expression changes were found in the CA1 or CA3 regions two weeks post-IH, although increased apoptosis in the CA1 region has been reported 1–2 days post-IH, returning to normal within two weeks [4]. The timing of responses may vary across brain regions. Spatially, the CA3 region has been shown to be more resilient to IH compared to the CA1 region in adult rats [7], highlighting different sensitivities to IH across brain regions.

One potential explanation for the region- and sex-specific effects observed lies in the developmental stage of the animals. The male rats used here were at postnatal day 7, a period of rapid neurogenesis, synaptic formation, and circuit refinement. The DG exhibits heightened neurogenic activity and plasticity during early development [24]. Contrarily, a previous IH study used older animals (postnatal day 42) [12], with mature brain structures and reduced plasticity. The immature brain may possess compensatory mechanisms that buffer against IH-induced downregulation of BDNF, such as flexible modulation of neurotrophic factor expression or receptor sensitivity [25].

IH protocols may also account for discrepancies across studies. Here, animals were exposed to 20 cycles per hour of 4% oxygen, representing deep but less frequent hypoxic episodes. A prior study [12] used more frequent but milder hypoxia (e.g., 10% oxygen, 40 cycles per hour). Higher-frequency IH induces more oxidative stress and sustained inflammatory signaling, leading to greater suppression of BDNF [26]. Differences in analytical methods may also contribute to these discrepancies. A previous study [12] used Western blotting on whole hippocampal tissue, whereas we used region-specific immunohistochemistry. While immunostaining allows for localized assessment of protein expression, it may be less sensitive to subtle or post-translational modifications. Thus, exposure conditions and the resolution of analytic methods may account for the discrepancies in BDNF findings.

Despite the reduced memory, learning, and emotional development in behavioral tests, immunohistological data has shown that IH exposure elevates BDNF and BrdU levels in the DG and amygdala areas of male rats. BDNF is essential for post-stroke neural repair in the brain, including in the DG and amygdala [27]. A previous study using a rat ischemic stroke model showed that the BDNF levels in both hemispheres did not show an inverse correlation with the severity of neuronal death, regardless of the time elapsed since stroke onset [28]. This is because non-neuronal cells are also able to produce substantial amounts of BDNF in the brain after ischemic stroke, although unilateral ischemic stroke increases BDNF content in both hemispheres. Thus, an increased BDNF expression upon IH exposure may be a protective response of non-neuronal cells to IH-induced brain damage, similar to the increase in BDNF levels during cerebral ischemia. While there was almost no sex difference at the level of behavioral experiments, probably due to the restorative function in our infant IH rat models, there was a significant difference only in short-term memory between male IH and N groups, which is potently controlled by the DG area. This suggests that the increase in both the immunopositive area of BDNF and BrdU and the gene expression of *Ntrk2* and *Epas1* may be a protective response to tissue injury by IH.

*Ntrk2*, *Hif1a*, and *Epas1* were used to comprehensively evaluate neuronal responses and compensatory mechanisms in the hippocampus and amygdala following IH exposure.

*Ntrk2* encodes TrkB, the high-affinity receptor for BDNF. The BDNF–TrkB signaling pathway plays a critical role in synaptic plasticity, neurogenesis, and the formation of memory and learning processes [29]. Changes in *Ntrk2* expression may serve as a useful indicator of neuroprotective or compensatory responses induced by IH.

*Hif1a* encodes hypoxia-inducible factor 1-alpha (HIF-1α), a key transcription factor that mediates acute to subacute cellular responses to hypoxic conditions. HIF-1α induces the expression of neuroprotective genes such as VEGF, erythropoietin, and antioxidant enzymes under hypoxic stress [30]. It is suitable for monitoring the early responses to acute IH and serves as a molecular marker of oxygen sensing and compensatory adaptation in the brain.

*Epas1* encodes HIF-2α, which regulates more chronic and tissue-specific responses to hypoxia. Compared to HIF-1α, HIF-2α affects gene groups associated with neurogenesis, antioxidant stress responses, and angiogenesis [30]. Notably, HIF-2α interacts with the BDNF–TrkB pathway [31], suggesting a potential role in chronic hypoxia adaptation and long-term tissue remodeling.

These three genes represent a rational set of molecular indicators for multilayered evaluation of the cascade from environmental hypoxic stimuli to cellular detection (HIF-1α, HIF-2α), and subsequently to synaptic plasticity and neuroadaptation (Ntrk2/BDNF signaling). They explain how hypoxic stress in the brain translates into compensatory responses, ultimately affecting memory, learning, and emotional development.

We found increased expression of Ntrk2 and Epas1 in the DG of IH-exposed male rats. Therefore, even in the absence of significant BDNF upregulation, downstream signaling pathways may be activated via enhanced receptor availability or alternative ligand mechanisms. TrkB can be activated independently of BDNF by pharmacological agents, supporting the idea of receptor-level modulation as a key driver of plasticity [32]. Epas1 upregulation may reflect activation of hypoxia-responsive pathways involved in neuroprotection, angiogenesis, and metabolic adaptation [33]. Thus, the infant brain does not simply respond passively to IH stress but actively initiates compensatory mechanisms to preserve neural integrity, particularly in plastic regions such as the DG.

IH increased *Ntrk2* and *Hif1a* mRNA expression and BDNF protein expression in the amygdala of our male rat pups. While the response of the amygdala to IH in rat pups has not been previously investigated, evidence suggests that the amygdala and hippocampus are vulnerable to hypoxic stress due to inflammation [34]. These findings imply that IH impairs emotional development in infant rats by inducing inflammation and hypoxic stress in the amygdala.

The current study demonstrated that only male infants exhibited elevated gene expression of *Ntrk2*, *Hif1a*, and *Epas1* in the DG and amygdala areas, with significant increases in BrdU and BDNF immunopositive areas. The sex-dependent effects of IH may be attributed to the sexual dimorphism of microglial cells in the central nervous system [35], which is essential for neuronal connections, neurogenesis during development, and the immune response to nerve injury. Microglia exhibit significant sexual dimorphism during neonatal development and are crucial for masculinization of the male brain [36]. Neonatal hypoxic-ischemic encephalopathy, a leading cause of motor and cognitive impairments in children, is largely initiated through microglial activation. Similarly, in a mouse model of neonatal hypoxic-ischemic encephalopathy, male neonates had more activated microglial cells than female neonates. Therefore, microglial activation, mobilization, and leukocyte infiltration may cause secondary nerve damage following IH-induced injury. Additionally, microglia provide growth factors and aid in nerve repair [37]. Factors such as animal age, hypoxia severity, and the timing of post-exposure observations may influence these microglial effects.

In this study, males were more strongly affected by IH, contrasting with previous studies [35] where females were more affected, possibly due to the timing of hypothalamic-pituitary-adrenal (HPA) axis maturation, responsible for organismal homeostasis. The difference may be attributed to the use of younger rats (up to 3 weeks old) in this study, compared to previous experiments [35]. In rats, crucial developmental stages occur between 1–60 days, including androgen imprinting, HPA axis maturation, and the emergence of HPA axis sexual dimorphism [38]. The stronger IH effect in males may be explained by the study's timing before the HPA axis's sexual differentiation, which occurs at 30–60 days [38].

In conclusion, this is the first study to reveal that IH affects specific subregions of the hippocampus and amygdala in infant rats, leading to memory/learning deficits and increased anxiety. Males exhibited greater susceptibility to ischemic insults and long-term deficits compared to females, emphasizing sex- and region-specific hippocampal vulnerability. Further research should investigate multiple time points to assess more comprehensive feedback mechanisms.

## References

[1] Zaffanello M, Ferrante G, Zoccante L et al (2023) Predictive power of oxygen desaturation index (ODI) and apnea-hypopnea index (AHI) in detecting long-term neurocognitive and psychosocial outcomes of sleep-disordered breathing in children: a questionnaire-based study. J Clin Med 12:3060. 10.3390/jcm12093060 37176501 10.3390/jcm12093060 PMC10179379

[2] Badran M, Gozal D (2025) Intermittent hypoxia as a model of obstructive sleep apnea: present and future. Sleep Med Clin 20:93–102. 10.1016/j.jsmc.2024.10.009 39894602 10.1016/j.jsmc.2024.10.009 PMC11788578

[3] Li H, Chen L, Wu X et al (2022) The effects of obstructive sleep apnea-hypopnea syndrome (OSAHS) on learn and memory function of 6–12 years old children. Int J Pediatr Otorhinolaryngol 159:111194. 10.1016/j.ijporl.2022.111194 35709564 10.1016/j.ijporl.2022.111194

[4] Gozal D, Daniel JM, Dohanich GP (2001) Behavioral and anatomical correlates of chronic episodic hypoxia during sleep in the rat. J Neurosci 21:2442–2450. 10.1523/JNEUROSCI.21-07-02442.2001 11264318 10.1523/JNEUROSCI.21-07-02442.2001 PMC6762394

[5] Gozal E, Row BW, Schurr A, Gozal D (2001) Developmental differences in cortical and hippocampal vulnerability to intermittent hypoxia in the rat. Neurosci Lett 305:197–201. 10.1016/s0304-3940(01)01853-5 11403939 10.1016/s0304-3940(01)01853-5

[6] Alkadhi KA (2019) Cellular and molecular differences between area CA1 and the dentate gyrus of the hippocampus. Mol Neurobiol 56:6566–6580. 10.1007/s12035-019-1541-2 30874972 10.1007/s12035-019-1541-2

[7] Gozal E, Gozal D, Pierce WM et al (2002) Proteomic analysis of CA1 and CA3 regions of rat hippocampus and differential susceptibility to intermittent hypoxia. J Neurochem 83:331–345. 10.1046/j.1471-4159.2002.01134.x 12423243 10.1046/j.1471-4159.2002.01134.x

[8] Xie H, Yung WH (2012) Chronic intermittent hypoxia-induced deficits in synaptic plasticity and neurocognitive functions: a role for brain-derived neurotrophic factor. Acta Pharmacol Sin 33:5–10. 10.1038/aps.2011.184 22212429 10.1038/aps.2011.184 PMC4010262

[9] Schirò G, Iacono S, Ragonese P et al (2022) A brief overview on BDNF-Trk pathway in the nervous system: a potential biomarker or possible target in treatment of multiple sclerosis? Front Neurol 13:917527. 10.3389/fneur.2022.917527 35911894 10.3389/fneur.2022.917527 PMC9332890

[10] Ogawa T, Okihara H, Kokai S et al (2018) Nasal obstruction during adolescence induces memory/learning impairments associated with BDNF/TrkB signaling pathway hypofunction and high corticosterone levels. J Neurosci Res 96:1056–1065. 10.1002/jnr.24216 29392750 10.1002/jnr.24216

[11] Okihara H, Ito J, Kokai S et al (2014) Liquid diet induces memory impairment accompanied by a decreased number of hippocampal neurons in mice. J Neurosci Res 92:1010–1017. 10.1002/jnr.23383 24687840 10.1002/jnr.23383

[12] Xie H, Leung KL, Chen L et al (2010) Brain-derived neurotrophic factor rescues and prevents chronic intermittent hypoxia-induced impairment of hippocampal long-term synaptic plasticity. Neurobiol Dis 40:155–162. 10.1016/j.nbd.2010.05.020 20553872 10.1016/j.nbd.2010.05.020

[13] Haukvik UK, McNeil T, Lange EH et al (2014) Pre- and perinatal hypoxia associated with hippocampus/amygdala volume in bipolar disorder. Psychol Med 44:975–985. 10.1017/S0033291713001529 23803260 10.1017/S0033291713001529 PMC3936825

[14] Cerullo MA, Adler CM, Delbello MP, Strakowski SM (2009) The functional neuroanatomy of bipolar disorder. Int Rev Psychiatry 21:314–322. 10.1080/09540260902962107 20374146 10.1080/09540260902962107

[15] Carty ML, Wixey JA, Kesby J et al (2010) Long-term losses of amygdala corticotropin-releasing factor neurons are associated with behavioural outcomes following neonatal hypoxia-ischemia. Behav Brain Res 208:609–618. 10.1016/j.bbr.2010.01.007 20085787 10.1016/j.bbr.2010.01.007

[16] Baum DM, Saussereau M, Jeton F et al (2018) Effect of gender on chronic intermittent hypoxic Fosb Expression in cardiorespiratory-related brain structures in mice. Front Physiol 9:788. 10.3389/fphys.2018.00788 29988603 10.3389/fphys.2018.00788 PMC6026892

[17] Aubrecht TG, Jenkins R, Magalang UJ, Nelson RJ (2015) Influence of gonadal hormones on the behavioral effects of intermittent hypoxia in mice. Am J Phys Regul Integr Comp Phys 308:R489–R499. 10.1152/ajpregu.00379.2014 10.1152/ajpregu.00379.2014 PMC4360067 25552660

[18] Stratilov V, Potapova S, Safarova D et al (2024) Prenatal hypoxia triggers a glucocorticoid-associated depressive-like phenotype in adult rats, accompanied by reduced anxiety in response to stress. Int J Mol Sci 25:5902. 10.3390/ijms25115902 38892090 10.3390/ijms25115902 PMC11172361

[19] Nagai H, Tsuchimochi H, Yoshida K, Shirai M, Kuwahira I (2014) A novel system including an N2 gas generator and an air compressor for inducing intermittent or chronic hypoxia. Int J Clin Exp Physiol 1:307–310

[20] Maeda H, Nagashima E, Hayashi YK, Kikura-Hanajiri R, Yoshida K (2018) MDMB-CHMICA induces thrashing behavior, bradycardia, and slow pressor response in a CB1- and CB2-receptor-dependent manner in conscious rats. Forensic Toxicol 36:313–319. 10.1007/s11419-018-0405-1

[21] Lumeng JC, Chervin RD (2008) Epidemiology of pediatric obstructive sleep apnea. Proc Am Thorac Soc 5:242–252. 10.1513/pats.200708-135MG 18250218 10.1513/pats.200708-135MG PMC2645255

[22] Leuner B, Gould E (2010) Structural plasticity and hippocampal function. Annu Rev Psychol 61:111–140. 10.1146/annurev.psych.093008.100359 19575621 10.1146/annurev.psych.093008.100359 PMC3012424

[23] Berdugo-Vega G, Dhingra S, Calegari F (2023) Sharpening the blades of the dentate gyrus: how adult-born neurons differentially modulate diverse aspects of hippocampal learning and memory. EMBO J 42:e113524. 10.15252/embj.2023113524 37743770 10.15252/embj.2023113524 PMC11059975

[24] Ding Y, Li L, Wang S et al (2023) Electroacupuncture promotes neurogenesis in the dentate gyrus and improves pattern separation in an early Alzheimer’s disease mouse model. Biol Res 56:65. 10.1186/s40659-023-00472-z 38041203 10.1186/s40659-023-00472-z PMC10693055

[25] Cancedda L, Putignano E, Sale A et al (2004) Acceleration of visual system development by environmental enrichment. J Neurosci 24:4840–4848. 10.1523/JNEUROSCI.0845-04.2004 15152044 10.1523/JNEUROSCI.0845-04.2004 PMC6729456

[26] Lee MYK, Ge G, Fung ML et al (2018) Low but not high frequency of intermittent hypoxia suppresses endothelium-dependent, oxidative stress-mediated contractions in carotid arteries of obese mice. J Appl Physiol 125:1384–1395. 10.1152/japplphysiol.00224.2018 30091668 10.1152/japplphysiol.00224.2018

[27] Yilmaz E, Acar G, Onal U et al (2024) Effect of 2-week naringin supplementation on neurogenesis and BDNF levels in ischemia-reperfusion model of rats. NeuroMolecular Med 26:4. 10.1007/s12017-023-08771-0 38457013 10.1007/s12017-023-08771-0 PMC10924031

[28] Béjot Y, Prigent-Tessier A, Cachia C et al (2011) Time-dependent contribution of non neuronal cells to BDNF production after ischemic stroke in rats. Neurochem Int 58:102–111. 10.1016/j.neuint.2010.10.019 21074587 10.1016/j.neuint.2010.10.019

[29] Miranda M, Morici JF, Zanoni MB et al (2019) Brain-derived neurotrophic factor: a key molecule for memory in the healthy and the pathological brain. Front Cell Neurosci 13:363. 10.3389/fncel.2019.00363 31440144 10.3389/fncel.2019.00363 PMC6692714

[30] Bakleh MZ, Al Haj Zen A (2025) The distinct role of HIF-1α and HIF-2α in hypoxia and angiogenesis. Cells 14:673. 10.3390/cells14090673 40358197 10.3390/cells14090673 PMC12071368

[31] Scott AL, Zhang M, Nurse CA (2015) Enhanced BDNF signalling following chronic hypoxia potentiates catecholamine release from cultured rat adrenal chromaffin cells. J Physiol 593:3281–3299. 10.1113/JP270725 26095976 10.1113/JP270725 PMC4553053

[32] Rantamäki T, Vesa L, Antila H et al (2011) Antidepressant drugs transactivate TrkB neurotrophin receptors in the adult rodent brain independently of BDNF and monoamine transporter blockade. PLoS One 6:e20567. 10.1371/journal.pone.0020567 21666748 10.1371/journal.pone.0020567 PMC3110188

[33] Oktay Y, Dioum E, Matsuzaki S et al (2007) Hypoxia-inducible factor 2alpha regulates expression of the mitochondrial aconitase chaperone protein frataxin. J Biol Chem 282:11750–11756. 10.1074/jbc.M611133200 17322295 10.1074/jbc.M611133200

[34] Rybnikova E, Nalivaeva N (2021) Glucocorticoid-dependent mechanisms of brain tolerance to hypoxia. Int J Mol Sci 22:7982. 10.3390/ijms22157982 34360746 10.3390/ijms22157982 PMC8348130

[35] Netto CA, Sanches E, Odorcyk FK, Duran-Carabali LE, Weis SN (2017) Sex-dependent consequences of neonatal brain hypoxia-ischemia in the rat. J Neurosci Res 95:409–421. 10.1002/jnr.23828 27870406 10.1002/jnr.23828

[36] Lenz KM, Nugent BM, Haliyur R, McCarthy MM (2013) Microglia are essential to masculinization of brain and behavior. J Neurosci 33:2761–2772. 10.1523/JNEUROSCI.1268-12.2013 23407936 10.1523/JNEUROSCI.1268-12.2013 PMC3727162

[37] Jin R, Yang G, Li G (2010) Inflammatory mechanisms in ischemic stroke: role of inflammatory cells. J Leukoc Biol 87:779–789. 10.1189/jlb.1109766 20130219 10.1189/jlb.1109766 PMC2858674

[38] Toews JNC, Hammond GL, Viau V (2021) Liver at the nexus of rat postnatal HPA axis maturation and sexual dimorphism. J Endocrinol 248:R1–R17. 10.1530/JOE-20-0286 33112814 10.1530/JOE-20-0286

